# Non-alcoholic fatty liver disease in Africa: a hidden danger

**DOI:** 10.1017/gheg.2019.2

**Published:** 2019-04-12

**Authors:** Imran M. Paruk, Fraser J. Pirie, Ayesha A. Motala

**Affiliations:** Department of Diabetes and Endocrinology, University of KwaZulu-Natal, Nelson R Mandela School of Medicine, Durban, South Africa

**Keywords:** Africa, non-alcoholic fatty liver disease, non-alcoholic steatohepatitis, obesity, type-2 diabetes

## Abstract

There is a dearth of data on the burden and spectrum of non-alcoholic fatty liver disease (NAFLD) in African populations. The limited available information suggests that the prevalence of NAFLD in the general population is lowest for the Africa region. However, this is likely to be an underestimate and also does not take into consideration the long-term impact of rising rates of obesity, type-2 diabetes mellitus (T2DM) and high human immunodeficiency virus infection burden in Africa. A racial disparity in the prevalence of NAFLD has been observed in some studies but remains unexplained. There is an absence of data from population-based studies in Africa and this highlights the need for such studies, to reliably define the health service needs for this region. Screening for NAFLD at a population-based level using ultrasound is perhaps the ideal method for resource-poor settings because of its relative cost-effectiveness. What is required as a priority from Africa, are well-designed epidemiologic studies that screen for NAFLD in the general population as well as high-risk groups such as patients with T2DM or obesity.

## Introduction

Non-alcoholic fatty liver disease (NAFLD) is defined as the presence of hepatic steatosis in the absence of excessive alcohol intake and it encompasses a wide range of histologic manifestations from simple steatosis, non-alcoholic steatohepatitis (NASH) and/or fibrosis to cirrhosis. Although simple steatosis follows a more benign course, NASH is typified by invasion of inflammatory cells into the hepatic parenchyma and has potential to progress to cirrhosis which poses a risk of hepatocellular carcinoma (HCC) [1, 2]. Disease progression with NASH is usually slow and spans many decades but a higher overall mortality and liver-related death has been reported [2, 3]. There is a dearth of information on the burden and spectrum of NAFLD in African populations, both from population-based and clinical studies. The limited available information suggests that the prevalence of NAFLD in the general population is lowest for the Africa region but is likely to be an underestimation. On the other hand, small clinic-based studies report variable prevalence. Taking into consideration the impact of rising obesity and type-2 diabetes mellitus (T2DM) prevalence in Africa, the burden of NAFLD is expected to increase [4]. However, there is an absence of data from population-based studies in Africa and this highlights the need for such studies to reliably define the health service needs for this region.

## Burden of NAFLD and the evidence from Africa

The actual prevalence of NAFLD may vary depending on the sensitivity of the detection method used. Whilst liver biopsy remains the gold standard to establish the diagnosis and grade severity, it is limited by its inherent risks and impracticality. Therefore, non-invasive radiologic imaging remains the most useful method of detecting liver steatosis. In a recent meta-analysis of studies using imaging or liver biopsy for diagnosis, the regional prevalence of NAFLD in the world was estimated to be 23.4% in Asia, 23.7% in Europe, 31.8% in the Middle-East, 24.1% in North America, 30.5% in South America and 13.5% in Africa [4]. The reported prevalence of NAFLD from population-based studies in high-income countries (HIC) such as the United States, is 8.1% based on elevated transaminase levels, 18.8% using ultrasound (USS) for diagnosis and 31% with magnetic resonance (MR) spectroscopy [5–7]. The prevalence in HIC is increasing with the worsening obesity and T2DM epidemics [8]. It is interesting to note that the region with the lowest reported prevalence was Africa (13.48%) [6]. However, the meta-analysis only included two studies from Africa with a small sample size. Using USS, Almobarak *et al*. found NAFLD in 20% of 100 asymptomatic Sudanese subjects and Onyekwere *et al*. reported a prevalence of 4.5% among 44 Nigerians [9, 10] (Table 1). It is indeed questionable whether these data can be extrapolated to all countries in Africa and also highlights the paucity of population-based prevalence data from Africa.

**Table 1.** Prevalence of NAFLD in African studies

Country Author reference	Study population	*n*	Detection method	Prevalence (%)	Risk associations
Nigeria					
Onyekwere *et al*. [10]	Control subjects^a^	44	USS	4.5	
	T2DM	106	USS	9.5	WC, ALT
Olusanya *et al*. [13]	Control subjects^b^	168	USS	1.2	
	T2DM	168	USS	16.7	WC^c^, BMI^c^, HDL^c^
Afolabi *et al*. [14]	T2DM	80	USS	68.8	BMI, HbA_1c_
Sudan					
Almobarak *et al*. [9]	Volunteers^d^	100	USS	20	
Almobarak *et al*. [15]	T2DM	167	USS	50.3	WC, BMI, HDL, Tg
Ethiopia					
Zawdie *et al*. [16]	T2DM	96	USS	73	ALT, Tg, Bilirubin
South Africa					
Kruger *et al*. [21]	OW or obese	127	Biopsy	87	

ALT, alanine transaminase; BMI, body mass index; HDL, high-density lipoprotein; HbA1c, haemoglobin A1c; NAFLD, nonalcoholic fatty liver disease; OW, overweight; Tg, total triglyceride; T2DM, type-2 diabetes; USS, ultrasound; WC, waist circumference.

a Non-diabetic age and sex matched.

b Non-diabetic sex-matched only.

c Male subjects only.

d Asymptomatic volunteers accompanying a patient.

Insulin resistance has been implicated in the pathogenesis of NAFLD and the current increasing burden of obesity and T2DM which are strongly associated with NAFLD, are believed to be the main drivers of this new scourge worldwide [4, 11, 12]. The prevalence of NAFLD among patients with T2DM is reported to be higher than the general population. A meta-analysis of 17 studies from 11 countries including one from Africa, reported a pooled prevalence of 54% [12]. There are limited data from Africa, with variable prevalence reported using USS, from 9.5% to 68.8% in Nigeria [10, 13, 14], 50.3% in Sudan [15] and 73% in Ethiopia [16] (Table 1). Elevated serum alanine aminotransferase, presumed to be due to NAFLD has been described in South African patients with T2DM; however other causes were not excluded [17]. Due to scanty data from a few African countries it is difficult to determine whether NAFLD prevalence in the general population or even among patients with T2DM in African countries mirror that described in other countries. The limited data suggest the prevalence of NAFLD in the general population is comparatively lower in Africa, but the prevalence in subjects with T2DM is similar to those reported globally.

NAFLD prevalence using USS or liver biopsy is reported to be higher (57.5–74%) in obese individuals [11]. NAFLD is considered to be a hepatic manifestation of the metabolic syndrome (MS) and in HIC the prevalence ranges from 47% to 71% in MS patients; MS also confers a higher risk of NASH and fibrosis [18, 19]. Visceral adipose tissue and its surrogate marker, waist circumference, have a stronger association with NAFLD than body mass index (BMI) alone and predispose to a greater risk of NASH and fibrosis [20]. The only African study to report on the prevalence of NAFLD among overweight or obese adults is from South Africa but was non-representative as participants were sourced from a hepatology clinic and only 5% were Black; in that study, of 127 patients who had liver biopsy, the prevalence of NAFLD, simple steatosis, NASH and advanced liver fibrosis was 87, 51, 36 and 17%, respectively [21].

Evidence for the impact of ethnicity on NAFLD comes from studies which showed that when compared to Hispanics or Whites, African Americans had a lower prevalence of NAFLD, despite having a greater burden of major risk factors for the disease [7, 22]. This racial disparity remains unexplained, but it has been speculated that the distribution of adiposity (predominantly subcutaneous rather than visceral among African Americans) may explain some of the observed differences [22]. Supportive evidence for this comes from a study of 106 female volunteers from South Africa which showed that African woman had a lower hepatic fat content on liver computed tomography (CT) scan compared to their Asian Indian and Caucasian counterparts, despite having a higher level of total body fat, subcutaneous body fat, BMI and waist circumference; moreover subcutaneous fat was found to be a significant negative determinant of hepatic fat content [23]. It has been proposed that subcutaneous fat serves as a storage depot for circulating triglycerides in the post-prandial state, thereby buffering the flux of free-fatty acids to the liver [24]. Therefore, visceral adipose tissue, which is associated with insulin resistance, may promote hepatic lipogenesis whilst subcutaneous fat stores may possibly be protective [23]. Other factors that may potentially explain the diversity in hepatic fat content across ethnic groups may include the degree of insulin resistance, dysglycaemia, socioeconomic factors that may influence dietary behaviour and genetic factors viz. patatin-like phospholipase domain-containing 3 (*PNPLA3*) gene [25].

Evidence from HIC indicates that human immunodeficiency virus (HIV)-infected patients on antiretroviral therapy (ART) have a high NAFLD prevalence (35%) using USS [26]. The available data for NAFLD among HIV infected patients in Africa are limited to two studies: a retrospective South African study which reported a prevalence of 28% on liver biopsy and a Nigerian cohort with a prevalence of 13.3% using USS [27, 28]. The Africa region has the highest burden of HIV infection world-wide and accounts for over two-thirds of the global total of new HIV infections [29]. UNAIDS Fast-Track approach to escalate access to ART to accomplish the 90–90–90 treatment target by 2020 may influence the prevalence of NAFLD in Africa [30]. Already, in eastern and southern Africa, ART use has escalated rapidly between 2010 and 2016 with over 10 million people accessing therapy [29]. The long-term implications of this rapid implementation of ART in low-income countries on NAFLD prevalence merit further investigation.

The healthcare agenda in sub-Saharan Africa has been dominated by poverty, undernutrition and communicable diseases with less attention paid to non-communicable diseases. However, with the epidemiological transition, rising obesity prevalence is reported from a number of African countries contributing to increasing prevalence of T2DM and other cardiometabolic diseases. From the limited evidence to date, it would appear that there is a high prevalence of NAFLD in T2DM in Africa, similar to that found in the West [13–16]. Therefore African nations are yet to experience the full force of this new epidemic and its consequences (NASH, cirrhosis and HCC).

## Unmet need for NAFLD data from Africa

A number of unmet needs exist with respect to NAFLD in Africa including the lack of epidemiologic data, the difficulty in explaining racial disparities observed and an incomplete understanding of the influence of body fat distribution on NAFLD prevalence. The potential impact of HIV and ART also requires investigation. What is required as a priority from Africa, are well-designed epidemiologic studies that screen for NAFLD in the general population as well as high-risk groups such as patients with T2DM or obesity. Screening for NAFLD at a population-based level using USS is perhaps the ideal method for resource-poor settings because of its relative cost-effectiveness, though it is limited by a lower sensitivity for detecting milder degrees of steatosis [31]. The use of elevated transaminase levels lacks sensitivity; MR- and CT-based imaging methods have better sensitivity but are costly. In view of the potential economic impact of NAFLD on health-care resources in Africa, it would be vital to determine the prevalence of fibrosis among these patients. In this regard, using transient elastography (TE) to measure liver stiffness as a surrogate marker of fibrosis, would be more acceptable than liver biopsy [32]. USS-based TE probes can now also quantify liver steatosis and together with the advantage of operator independent data collection and ability to detect fibrosis, makes TE an attractive option for large-scale studies. The UK Biobank MR imaging study has shown that quantifying hepatic fat using non-invasive MR scans in a large cohort is feasible and can provide accurate estimates of prospective risks of NAFLD [33]. Replication of large-scale initiatives such as this in Africa using MR or TE technology may provide a better understand of the epidemiology of NAFLD across this region.

## Conclusion

The reported prevalence for NAFLD in Africa in the general population is likely to be an underestimate. The long-term impact of rising obesity and T2DM prevalence in Africa makes NAFLD a hidden danger in this region. Health authorities in African countries need to be cognisant of this silent threat and develop strategic responses to deal with it by documenting its extent, adapting the national health plan and managing healthcare resources appropriately.

## References

[1] Teli MR, James OF, Burt AD, Bennett MK and Day CP (1995) The natural history of nonalcoholic fatty liver: a follow-up study. Hepatology 22, 1714–1719. 7489979

[2] Marrero J, Fontana RJ, Su GL, Conjeevaram HS, Emick DM and Lok AS (2002) NAFLD may be a common underlying liver disease in patients with hepatocellular carcinoma in the United States. Hepatology 36, 1349–1354. 12447858 10.1053/jhep.2002.36939

[3] Powell EE, Cooksley WG, Hanson R, Searle J, Halliday JW and Powell LW (1990) The natural history of nonalcoholic steatohepatitis: a follow-up study of forty-two patients for up to 21 years. Hepatology 11, 74–80. 2295475 10.1002/hep.1840110114

[4] Younossi ZM, Koenig AB, Abdelatif D, Fazel Y, Henry L and Wymer M (2016) Global epidemiology of nonalcoholic fatty liver disease-Meta-analytic assessment of prevalence, incidence, and outcomes. Hepatology 64, 73–84. 26707365 10.1002/hep.28431

[5] Ioannou GN, Boyko EJ and Lee SP (2006) The prevalence and predictors of elevated serum aminotransferase activity in the United States in 1999–2002. American Journal of Gastroenterology 101, 76–82. 16405537 10.1111/j.1572-0241.2005.00341.x

[6] Younossi ZM, Stepanova M, Negro F, Hallaji S, Younossi Y, Lam B, (2012) Nonalcoholic fatty liver disease in lean individuals in the United States. Medicine (Baltimore) 91, 319–327. 23117851 10.1097/MD.0b013e3182779d49

[7] Browning JD, Szczepaniak LS, Dobbins R, Nuremberg P, Horton JD, Cohen JC, Grundy SM and Hobbs HH, (2004) Prevalence of hepatic steatosis in an urban population in the United States: impact of ethnicity. Hepatology 40, 1387–1395. 15565570 10.1002/hep.20466

[8] Neuschwander-Tetri BA (2017) Non-alcoholic fatty liver disease. BMC Medicine 15, 45. 28241825 10.1186/s12916-017-0806-8 PMC5330146

[9] Almobarak AO, Barakat S, Khalifa MH, Elhoweris MH, Elhassan TM and Ahmed MH (2014) Non alcoholic fatty liver disease (NAFLD) in a Sudanese population: what is the prevalence and risk factors? Arab Journal of Gastroenterology 15, 12–15. 24630507 10.1016/j.ajg.2014.01.008

[10] Onyekwere CA, Ogbera AO and Balogun BO (2011) Non-alcoholic fatty liver disease and the metabolic syndrome in an urban hospital serving an African community. Annals of Hepatology 10, 119–124. 21502672

[11] Angulo P (2002) Nonalcoholic fatty liver disease. New England Journal of Medicine 346, 1221–1231. 11961152 10.1056/NEJMra011775

[12] Amiri Dash Atan N, Koushki M, Motedayen M, Dousti M, Sayehmiri F, Vafaee R, Norouzinia M and Gholami R (2017) Type 2 diabetes mellitus and non-alcoholic fatty liver disease: a systematic review and meta-analysis. Gastroenterology and Hepatology from Bed to Bench 10(Suppl. 1), S1–S7. 29511464 PMC5838173

[13] Olusanya TO, Lesi OA, Adeyomoye AA and Fasanmade OA (2016) Non alcoholic fatty liver disease in a Nigerian population with type II diabetes mellitus. Pan African Medical Journal 24, 1–9. 27583084 10.11604/pamj.2016.24.20.8181 PMC4992392

[14] Afolabi BI, Ibitoye BO, Ikem RT, Omisore AD, Idowu BM and Soyoye DO (2018) The relationship between glycaemic control and Non-alcoholic fatty liver disease in Nigerian type 2 diabetic patients. Journal of the National Medical Association 110, 256–264. 29778128 10.1016/j.jnma.2017.06.001

[15] Almobarak AO, Barakat S, Suliman EA, Elmadhoun WM, Mohamed NA, Abobaker IO, Noor SK, Bushara SO and Ahmed MH (2015) Prevalence of and predictive factors for nonalcoholic fatty liver disease in Sudanese individuals with type 2 diabetes: is metabolic syndrome the culprit? Arab Journal of Gastroenterology 16, 54–58. 26174761 10.1016/j.ajg.2015.06.001

[16] Zawdie B, Tadesse S, Wolide AD, Nigatu TA and Bobasa EM (2018) Non-Alcoholic fatty liver disease and associated factors among type 2 diabetic patients in Southwest Ethiopia. Ethiopian Journal of Health Sciences 28, 19–30. 29622904 10.4314/ejhs.v28i1.4 PMC5866286

[17] Paruk IM, Pirie FJ, Motala AA and Kolawole BA (2011) High prevalence of abnormal liver enzymes in South African patients with type 2 diabetes mellitus attending a diabetes clinic. Journal of Endocrinology, Metabolism and Diabetes of South Africa 16, 43–47.

[18] Marchesini G, Brizi M, Bianchi G, Tomassetti S, Bugianesi E, Lenzi M, Noor SK, Bushara SO and Ahmed MH (2001) Nonalcoholic fatty liver disease: a feature of the metabolic syndrome. Diabetes 50, 1844–1850. 11473047 10.2337/diabetes.50.8.1844

[19] Ryan MC, Wilson AM, Slavin J, Best JD, Jenkins AJ and Desmond PV (2005) Associations between liver histology and severity of the metabolic syndrome in subjects with nonalcoholic fatty liver disease. Diabetes Care 28, 1222–1224. 15855597 10.2337/diacare.28.5.1222

[20] Pang Q, Zhang J-Y, Song S-D, Qu K, Xu X-S, Liu S-S and Chang L (2015) Central obesity and nonalcoholic fatty liver disease risk after adjusting for body mass index. World Journal of Gastroenterology 21, 1650–1662. 25663786 10.3748/wjg.v21.i5.1650 PMC4316109

[21] Kruger F, Daniels C and Kidd M (2010) Non-alcoholic fatty liver disease (NAFLD) in the Western cape: a descriptive analysis. South African Medical Journal 100, 168–171. 20459941 10.7196/samj.1422

[22] Caldwell SH, Harris DM, Patrie JT and Hespenheide EE (2002) Is NASH underdiagnosed among African Americans? American Journal of Gastroenterology 97, 1496–1500. 12094872 10.1111/j.1572-0241.2002.05795.x

[23] Naran NH, Haagensen M and Crowther NJ (2018) Steatosis in South African women: how much and why? PLoS One 13, 1–12. 10.1371/journal.pone.0191388 PMC5774768 29351564

[24] Frayn K (2002) Adipose tissue as a buffer for daily lipid flux. Diabetologia 45, 1201–1210. 12242452 10.1007/s00125-002-0873-y

[25] Romeo S, Kozlitina J, Xing C, Pertsemlidis A, Cox D, Pennacchio LA, Boerwinkle E, Cohen JC and Hobbs HH (2008) Genetic variation in PNPLA3 confers susceptibility to nonalcoholic fatty liver disease. Nature Genetics 40, 1461–1465. 18820647 10.1038/ng.257 PMC2597056

[26] Maurice JB, Patel A, Scott AJ, Patel K, Thursz M and Lemoine M (2017) Prevalence and risk factors of nonalcoholic fatty liver disease in HIV-monoinfection. AIDS (London, England) 31, 1621–1632. 10.1097/QAD.0000000000001504 28398960

[27] Hoffmann CJ, Hoffmann JD, Kensler C, Van Watt MD, Omar T, Chaisson RE, Martinson NA and Variava E (2015) Tuberculosis and hepatic steatosis are prevalent liver pathology findings among HIV-infected patients in South Africa. PLoS One 10, 1–8. 10.1371/journal.pone.0117813 PMC4323253 25668620

[28] Lesi OA, Soyebi KS and Eboh CN (2009) Fatty liver and hyperlipidemia in a cohort of HIV-positive Africans on highly active antiretroviral therapy. Journal of the National Medical Association 101, 151–155. 19378632 10.1016/s0027-9684(15)30828-2

[29] Pustil R. (2016) Global AIDS. AIDS (London, England) 17(Suppl. 4), S3–S11. 15080170

[30] UNAIDS (2014) 90-90-90 An ambitious treatment target to help end the AIDS epidemic. Available at http://www.Unaids.Org/Sites/Default/Files/Media_Asset/90-90-90_En_0.Pdf.

[31] Bohte AE, van Werven JR, Bipat S and Stoker J (2011) The diagnostic accuracy of US, CT, MRI and ^1^H-MRS for the evaluation of hepatic steatosis compared with liver biopsy: a meta-analysis. European Radiology 21, 87–97. 20680289 10.1007/s00330-010-1905-5 PMC2995875

[32] Tapper EB and Loomba R (2018) Noninvasive imaging biomarker assessment of liver fibrosis by elastography in NAFLD. Nature Reviews. Gastroenterology & Hepatology 15, 274–282. 29463906 10.1038/nrgastro.2018.10 PMC7504909

[33] Wilman HR, Kelly M, Garratt S, Matthews PM, Milanesi M, Herlihy A, Gyngell M, Neubauer S, Bell JD, Banerjee R and Thomas EL (2017) Characterisation of liver fat in the UK Biobank cohort. PLOS ONE 12, e017686. 10.1371/journal.pone.0172921 PMC5328634 28241076

